# National survey of current practices and attitudes regarding discharge disposition for older adults with mild traumatic brain injury and traumatic intracranial hemorrhage

**DOI:** 10.1007/s13760-025-02834-8

**Published:** 2025-07-03

**Authors:** Juliette A. L. Santing, Robert Croese, Joukje van der Naalt, Heleen den Hertog, Korné Jellema

**Affiliations:** 1 Department of Neurology, University of Groningen, University Medical Center Groningen, PO Box 30.001, Groningen, 9700 RB The Netherlands; 2 Department of Neurology, Haaglanden Medical Center, PO Box 432, The Hague, 2501 CK The Netherlands; 3 Department of Neurology, PO 10400, 8000 GK, Isala, Zwolle, The Netherlands

**Keywords:** Mild traumatic brain injury, Traumatic intracranial hemorrhage, Older adults, Hospital admission, Discharge, Survey

## Abstract

**Background:**

Older patients with traumatic intracranial hemorrhage (tICH) following mild traumatic brain injury (mTBI) are commonly seen by neurologists in clinical practice. Due to the lack of clear evidence on optimal management, most guidelines recommend hospital admission for these patients. However, some studies have shown that selected low-risk patients can be safely discharged from the Emergency Department (ED). In light of these differing opinions and a lack of Class 1 evidence, we aimed to explore current management practices and attitudes toward tICH in older mTBI patients in the Netherlands.

**Methods:**

A national online survey, involving the disposition strategies for five case vignettes of tICH, was sent to neurology residents and neurologists to explore current variations in clinical practice. We evaluated patient and hemorrhage characteristics influencing decisions regarding the discharge disposition of a patient from the ED.

**Results:**

The survey was completed by 113 respondents, including 36 (32%) residents and 77 (68%) neurologists. In all the cases, over 70% of respondents preferred hospital admission over ED discharge for older mTBI patients with tICH. There was substantial variation in the respondents’ willingness to participate in a randomized trial evaluating the necessity of hospital admission after mTBI with tICH. Factors influencing admission varied between cases. A secondary deterioration risk of 1–2% was considered acceptable by the majority (53%) to allow direct ED discharge.

**Conclusion:**

Our findings demonstrate limited willingness among clinicians to discharge older mTBI patients with tICH directly from the ED. To support safe and consistent decision-making, high-quality evidence is urgently needed to guide disposition decisions for older mTBI patients with tICH.

**Supplementary Information:**

The online version contains supplementary material available at 10.1007/s13760-025-02834-8.

## Introduction

Mild traumatic brain injury (mTBI) is a common diagnosis in older adults presenting to the emergency departments (EDs) in Western countries [1]. In the Netherlands, the number of ED visits by older patients with TBI increased by more than 200% over the past decade, accompanied by an almost twofold rise in hospital admissions [2]. Older adults are at higher risk of traumatic intracranial hemorrhage (tICH) after mTBI, with reported rates of tICH reaching up to 21% in adults aged 65 years and older [3–6]. As the population continues to age, and a significant number of older individuals are taking antithrombotic medication [7], the incidence of tICH in older mTBI patients is expected to increase further [2, 8, 9].

Current national guidelines recommend hospital admission for all patients with tICH, regardless of the type, size, or location of the hemorrhage, or the clinical condition of the patient [10–12]. However, recent studies suggest that specific subgroups of mTBI patients may safely be discharged from the ED after a period of observation [13–16]. Currently, some hospitals in Europe, including the Netherlands, already discharge subgroups of mTBI patients with tICH directly from the ED with outpatient follow-up [17, 18]. Given the rapid increase in mTBI-related ED visits and the known negative effects of hospitalization on older adults [19–21], reducing hospital admissions in this population may offer several benefits. These include a reduction in hospital-acquired complications, such as nosocomial infections, falls, and medication errors, as well as decreased resource use and overall hospitalization costs.

To date, there are no clinically validated tools to reliably identify which patients with mTBI and accompanying tICH can be safely discharged home from the ED [22]. Likewise, prospective randomized studies aimed at identifying low-risk adult mTBI patients with tICH are lacking. Despite national guidelines recommending admission for mTBI patients with tICH [10], factors influencing admission decisions are notwell understood. Radiological and clinical abnormalities that reliably predict secondary deterioration have yet to be established. Additionally, it is unknown to what extent hospitals deviate from these guidelines by discharging subgroups of mTBI patients with tICH. To inform the design of a future randomized clinical trial, we aimed to explore current variations in management among neurologists and residents in the Netherlands, using an online questionnaire that included several CT characteristics of tICH. In addition, we assessed which demographic and injury-related characteristics influence treatment decisions.

## Methods

**Fig. 1:**
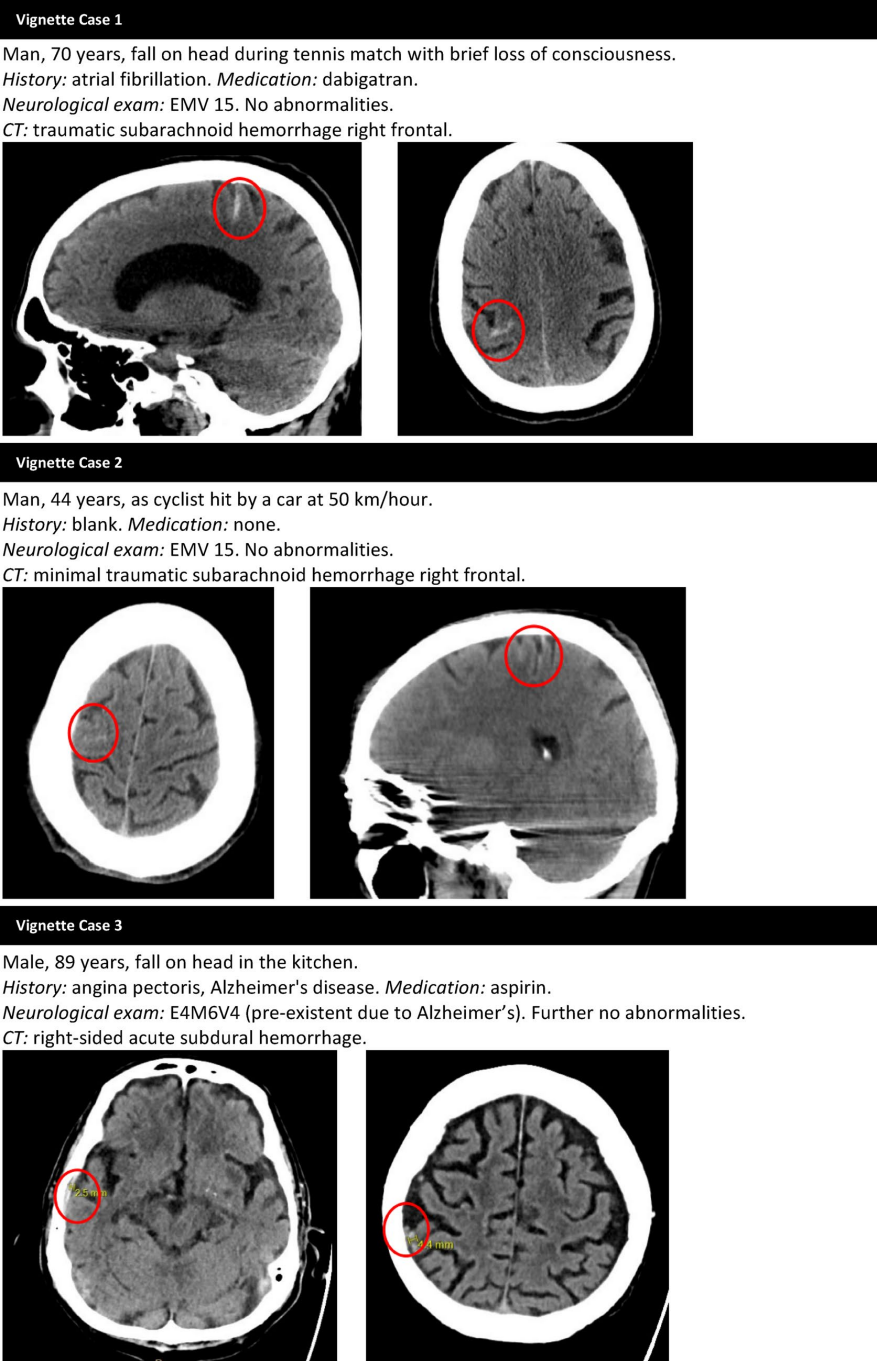

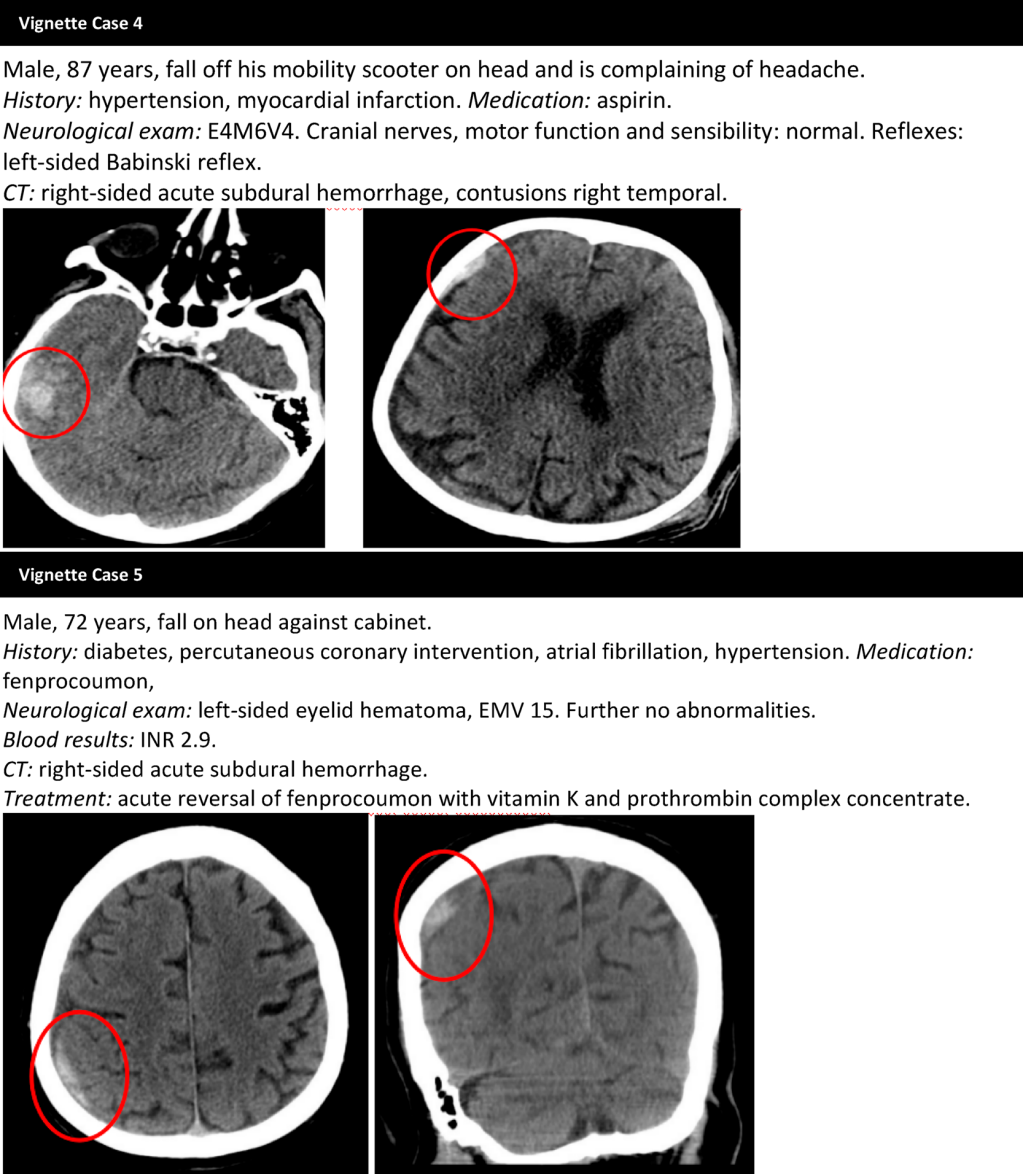
The five case vignettes and the accompanying CT scans

We conducted a national survey among neurology residents and neurologists using the web survey tool Survio (http://www.survio.com). The questionnaire consisted of three parts (Online Appendix 1). The first part collected demographic information about the respondents. Part two comprised questions about five separate mTBI cases (Fig. 1). We selected typical day-to-day cases of older adults with mTBI and tICH who had been seen at the ED of Haaglanden Medical Center. The cases were anonymized and varied in patient characteristics including age, Glasgow Coma Scale (GCS) score, type, size, and location of the tICH, use of antiplatelet or anticoagulant medication, and social circumstances. To examine the influence of age, we included one case involving an adult under 65 years. Respondents were asked whether they would admit the patients and which specific factors influenced their decisions. Additionally, they were asked if they would be willing to participate in a randomized clinical trial assessing the necessity of hospital admission for mTBI patients with tICH. The third part included a bonus question asking respondents to indicate what they considered an acceptable risk of secondary deterioration when discharging someone with tICH from the ED to home. The survey was open for responses between July 2024 and September 2024. The link of the survey was shared with residents (*n* = 337) and neurologists (*n* = 1246) through the newsletter of The Netherlands Society of Neurology and every teaching hospital (*n* = 15). A reminder was sent after two weeks, and the survey was closed two weeks later. All data were analyzed anonymously.

### Statistical analysis

We incorporated all received responses into our analysis. Descriptive statistics were used as appropriate. Statistical comparisons focused on work experience and practice variation, using the chi-square test, Fisher’s exact test, and Fisher-Freeman-Halton exact test as appropriate. Statistical analyses were performed using SPSS 28.0 (IBM, Chicago, IL, USA). P values < 0.05 were considered statistically significant.

A minimal statistical accuracy level of 15% with a 95% confidence level was considered to be acceptable [23]. We calculated the statistical accuracy of this survey based on population size and response rates from residents and neurologists. Higher response rates typically result in greater accuracy. Additionally, larger population sizes require fewer responses to achieve the same level of statistical accuracy as smaller populations [23, 24].

## Results

### Respondents’ characteristics

The survey was filled out by 113 respondents (7%), of whom 112 completed all questions. Of the respondents, 35 (31%) were residents and 78 (69%) were neurologists. Notably, differences between residents and neurologists were observed for age, gender, and type of department, properly reflecting the Dutch situation. The general characteristics of the respondents are listed in Table 1.

**Table 1:** Characteristics of the respondents

Responder’s characteristics	Residents (n=35)	Neurologists (n=78)
Male, no. (%)	9 (25.7)	31 (39.7)
Age group in years, no. (%)		
20-29	7 (20.0)	0 (0)
30–39	28 (80.0)	19 (24.4)
40–49	0 (0)	35 (44.9)
50–59	0 (0)	17 (21.8)
60–70	0 (0)	7 (9.0)
Years since finishing residency, no. (%)	-	
0-5		19 (24.4)
6-10		17 (21.8)
11-20		28 (35.9)
21-30		13 (16.7)
>30		1 (1.3)
Department, no. (%)		
Academic hospital	11 (31.4)	11 (14.1)
Top clinical teaching hospital	24 (68.6)	47 (60.3)
General hospital	0 (0)	20 (25.6)
Categorical hospital	0 (0)	0 (0)

### Accuracy

With a response of 7% from a population of 1583, the statistical accuracy of the survey was calculated at 9%, with a 95% confidence level.

### Management choices in five separate mTBI cases with tICH

Table 2 provides anoverview of the treatment decisions and the physicians’ willingness to randomize, based on five separate mTBI cases with tICH. While treatment choices varied, the majority of respondents chose to admit mTBI patients with tICH; specifically, 95%, 71%, 75%, 98%, and 98% answered “yes” to the question “Would you admit or not?” for each respective case. Willingness to randomize varied considerably, with 50%, 93%, 61%, 14%, and 82% of respondents expressing a positive response to randomization in each case.

**Table 2:** Questions, Possible Answers, and Responses (Proportions) with Regard to the Clinical Case Vignettes

Questions	Possible answers	Answers (%)				
		Case 1	Case 2	Case 3	Case 4	Case 5
Would you admit this patient?	Yes	107 (94.6)	80 (70.8)	85 (75.2)	111 (98.2)	111 (98.2)
	No	5 (4.4)	26 (23.0)	22 (19.5)	1 (0.9)	2 (1.8)
	Other*	1 (0.9)	7 (6.2)	6 (5.3)	1 (0.9)	0 (0)
Would you be willing to leave this decision (whether to admit or not) open for randomization in a study?	Yes	57 (50.4)	105 (92.9)	69 (61.1)	16 (14.2)	93 (82.3)
	No	54 (47.8)	8 (7.1)	39 (34.5)	96 (85.0)	20 (17.7)
	Other*	2 (1.8)	0 (0)	5 (4.4)	1 (0.9)	0 (0)

*Other reasons are shown in Online Appendix 2

### Influencing factors

**Fig. 2:**
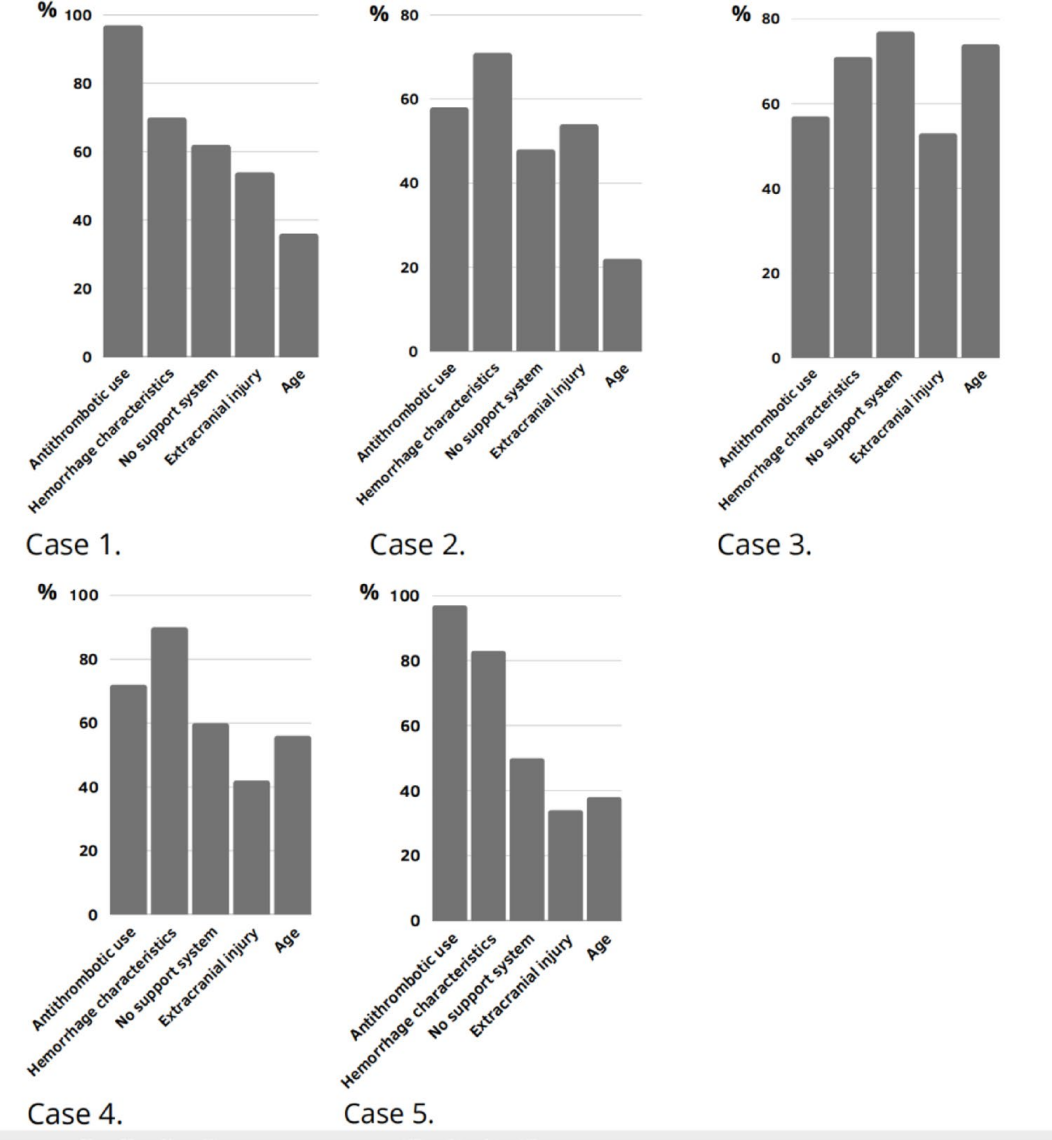
Factors influencing decision-making per case

Although the influencing factors for the decision to admit varied per case (Fig. 2), antithrombotic use and hemorrhage characteristics were most frequently cited as key considerations. The physicians’ attitude towards admission was not related to the years or type of practice (*Online Appendix 3*). However, respondents with more clinical experience appeared more likely to consider extracranial injuries or the absence of a support system as factors influencing the decision to admit (*Online Appendix 4*).

### Acceptable risk of secondary deterioration

More than half of the respondents (53%) considered a 1–2% risk of secondary deterioration to be acceptable when discharging a patient with tICH from the ED to home. An additional 35% accepted a 3–5% risk, and 5% accepted a 6–10% risk. 7% of respondents believed that all mTBI patients with tICH should be admitted to the hospital for observation.

## Discussion

Our nationwide survey provides the first data on current emergency department (ED) discharge and admission decisions for older adults with mild traumatic brain injury (mTBI) patients and traumatic intracranial hemorrhage (tICH). The majority of respondents preferred hospital admission in older mTBI patients with tICH. Responses to the case vignettes suggested that the willingness to randomize patients in a trial evaluating the necessity of hospital admission for mTBI with tICH was influenced by factors such as antithrombotic use, hemorrhage characteristics, and pre-existent cognitive function. Physicians demonstrated a low tolerance for secondary deterioration risk, with over half (53%) considering a 1–2% risk acceptable for direct discharge from the ED.

The case vignettes in this survey reflect common situations encountered in the ED. Both residents and neurologists are often confronted with a heterogeneous population of older mTBI patients with tICH, and disposition decisions can be challenging. Besides the potential health benefits, it also comes with high costs, significant resource use [25], and an increased risk of adverse events, such as cognitive decline, infections, and deconditioning [19–21]. Despite these considerations, the findings of this survey indicate that hospital admission remains the most widely chosen and generally undisputed management strategy for older mTBI patients with tICH.

Why do neurologists in this context opt for hospital admission? Age is the key determinant impacting outcomes after TBI with tICH [26]. Older adults frequently present with comorbidities [27–29] and use antithrombotic medications [30]– [31], both linked to worse clinical outcomes. Additionally, subdural hemorrhage—a common form of tICH in this population—is associated with greater risks [32–34]. Given these risk factors, hospital admission is an intuitive and cautious choice from a clinical standpoint. Nevertheless, many older adults with TBI and tICH recover well and uneventfully. Chronological age and TBI severity alone are inadequate prognostic markers; preinjury function, comorbidities, and frailty are believed to be more predictive of outcomes after TBI [1]. Currently, there is a lack of guidance on which older patients with tICH are safe for direct ED discharge [10–12]. Two clinical tools have been developed to predict which mTBI patients with tICH can be safely discharged home [17, 35]. However, a retrospective comparison found that less than 2% of tICH patients would have been deemed safe for ED discharge using either model [36]. Therefore, these tools offer limited added value in influencing or changing clinical decision-making for mTBI patients with tICH in the current practice. Moreover, these models are often not applicable to older patients, as criteria precluding discharge (e.g., age, antithrombotic medication, comorbidities) are often present in this population. As a result, applying these tools may lead to a substantial increase in hospital admissions, while the actual number of patients who experience clinical deterioration remains relatively low.

We identified per-case patient- and injury-related factors that influenced the decision to admit to the hospital or ward. Many of these factors were expected, such as higher admission rates for patients using antithrombotic medication or those with a large tICH, both of which are associated with adverse outcomes [37]. Nonetheless, we observed that the decision to admit a mTBI patient with tICH is not solely determined by the presence of high-risk factors. Around 70% of the respondents in our study favored hospital admission for a younger adult with minimal tSAH, both factors associated with a low risk of secondary deterioration [15, 16]. This suggests that beyond demographic and injury-related factors, other element contribute to the decision to admit a mTBI patient with tICH. A previous study found that over half of admissions are influenced by nonclinical factors [38]. For instance, admissions may also occur for poor social circumstances, family opinions or inability to provide outpatient follow-up care [17, 38, 39]. Low risk tolerance and fear of malpractice suits are additional factors associated with increased defensive medical practices and a greater tendency to admit patients [40–42]. In our survey, care providers’ attitudes towards hospital admission were not related to years of experience or type of hospital practice. However, there was a trend that respondents with more work experience were more inclined to admit patients with extracranial injuries or lacking support systems. This could reflect their experience that these factors are associated with unsuccessful outpatient management leading to negative outcomes. In accordance with a previous study [43], we found that the risk tolerance for secondary deterioration among residents and neurologists is low. Secondary deterioration after mTBI with tICH can have devastating consequences for patients. In such a case, no one wants to be sued on the premise of not providing maximal care and not least because of guideline-discordant care [10–12]. To prevent unnecessary hospital admission of older patients, future TBI guidelines should provide more specific guidance on disposition decisions for mTBI patients with tICH. Further, guidelines may need to take into account a range of neurologists’ risk tolerance preferences regarding direct ED discharge of low risk mTBI patients with tICH or even take patient referral into account. Further studies are needed to determine whether the implementation of such guidelines can reduce hospital admission of low risk mTBI patients with tICH.

Given the aging population in Western countries and the significant economic burden of TBI, prediction tools and guidelines specifically tailored to the management of older mTBI patients with tICH would be highly valuable for physicians in the ED. The strong preference for hospital admission in these patients reflects the lack of high-quality evidence and the need for high-quality studies on disposition strategies. The variation in willingness to randomize older mTBI patients with tICH in a trial assessing the necessity of hospital admission makes a randomized controlled trial challenging to implement. Therefore, a prospective, observational, cluster-based, parallel-group study could be a more effective approach, as it would allow for evaluation of current practices. This design is expected to result in greater participation and a higher enrollment rate to address a growing clinical and societal problem.

### Strengths and limitations

The strengths of our study include being the first to address this issue and the inclusion of respondents from all types of hospitals. In daily clinical practice, neurologists must make treatment decisions for older mTBI patients with tICH during the acute phase. Our survey was designed as a case-based questionnaire, with cases that differed regarding clinical and hemorrhage characteristics, to reflect daily clinical practice. Additionally, we assessed factors influencing disposition decisions and investigated physicians’ risk tolerance regarding the discharge of mTBI patients with tICH from the ED.

This study also has several limitations. First, although this online survey reflects real-life clinical settings, but respondents may have found it difficult to make recommendations due to the lack of opportunity to request further details or to have full knowledge of the clinical subtleties. Second, the response rate was relatively low; however, the diversity of the respondents is more relevant to the objectives of this study. Rather than focusing on individual practices, our aim was to capture variation in clinical decision-making across different institutional settings. The respondents represented a broad spectrum of hospitals, levels of clinical experience, and age groups, which supports the generalizability of our results across different institutional practices. Furthermore, low response rates do not necessarily compromise the accuracy of survey measurements [43]. Importantly, our response rate is comparable to that of previous surveys on acute neurological disorders among neurologists in the Netherlands [44–46]. Nevertheless, we acknowledge that some caution is warranted when generalizing our results to the broader population of residents and neurologists in the Netherlands. Third, response bias may have affected our results, as the survey may have attracted only those specialists with a particular interest in this topic. Finally, given differences between healthcare systems, the results may not be generalizable to other countries.

## Conclusion

This survey shows that, in the Netherlands, neurologists generally favor hospital admission for patients with mTBI and tICH, and that they have a low tolerance for the risk of secondary deterioration. Attitudes toward randomizing patients in a trial to assess the necessity of hospital admission were mixed. The findings indicate that, at present, there is insufficient support for the safe discharge of selected older mTBI patients with tICH directly from the ED. High-quality evidence is needed to identify which of these patients, who are currently admitted, could potentially be managed safely in an outpatient setting, thereby reducing unnecessary hospital admissions.

## Electronic supplementary material

Below is the link to the electronic supplementary material.

Supplementary Material 1

## Data Availability

No datasets were generated or analysed during the current study.
