# Supplementary material for: National survey of current practices and attitudes regarding discharge disposition for older adults with mild traumatic brain injury and traumatic intracranial hemorrhage
Source: Acta Neurol Belg. 2025 Jul 3;125(4):1073–81. doi: 10.1007/s13760-025-02834-8 (PMC12391191; doi:10.1007/s13760-025-02834-8)
Supplement: Supplementary file 1 — Supplementary Material 1 [file 13760_2025_2834_MOESM1_ESM.docx]

*Appendix 1: overview questionnaire.*

| **Part 1:**  **Respondent characteristics** | *Age group* -20-29 years -30-39 years -30-49 years  -50-59 years -60-70 years |
| --- | --- |
|  | *Sex* -Male -Female |
|  | *Function* -Neurologist -Resident |
|  | *Years since finishing residency* -0-5 years -6-10 years -11-20 years -21-30 years  ->30 years |
|  | *Practice -*Academic hospital -Top clinical teaching hospital -General hospital -Specialized hospital |
| **Part 2: Treatment questions** | *Would you admit this patient?*  -Yes  -No |
|  | *Would you be willing to leave this decision (whether to admit or not) open for randomization in a study?*  -Yes  -No |
| **Part 3: Factors influencing treatment decisions** | *Which of the following factors influence the decision to admit?* -Antithrombotic use -Hemorrhage characteristics -No support system -Extracranial injury -Age |
| **Part 4: Bonus question** | *What do you consider to be an acceptable risk of secondary deterioration to discharge someone with mTBI and tICH instead of admitting them?* -1-2% -3-5% -6-10%  -Every mTBI patients with tICH should be admitted |

*Appendix 2: Other reasons for admitting or randomizing.*

Admission

*Case 1 (n=1)*

-If he randomizes for dismissal I would find this very disconcerting when using a DOAC

*Case 2 (n=7)*-According to the current protocol, yes, but I am more inclined to deviate from the protocol if there are no neurological deficits

-Sometimes when still having PTA or presentation in the night

-Depending on bed capacity

-Supervisor probably want to admit

-Depends on the time of day and whether the radiologist has already assessed the CT

-Depends on social support system

-Depends on additional factors

*Case 3 (n=6)*

-Depending on the family’s opinion
-Depending on the family’s opinion and wish for a neurosurgical intervention if necessary (n=2)

-Depends on social support system

-Insufficient data available

-Depending on the risk of delirium / whether or not surgery is required in case of deterioration
-In doubt, very small SDH. No social support system?

*Case 4 (n=1)*

-The question is what the benefit is of a CT. We know for sure that it has no treatment consequences

Randomization

*Case 1 (n=2)*

-Depending on the study protocol and the substantiation
-Yes, but also depending on the time of taking dabigatran and the possibility of stopping this and whether the indication for continuation is harder than that of discontinuation

*Case 3 (n=5)*-Depending on the social support system (n=2)
-Alzheimer patients should be excluded for studies like this

-The question is what the benefit is of a CT. We know for sure that it has no treatment consequences
-If he randomizes for discharge, that wouldn't go anyway

*Case 4 (n=1)
-*I find it difficult to leave this to a trial, there is a real chance of deterioration

*Appendix 3: The Relationship between the respondent’s (A) work experience and (B) practice and tendency to admit.*

**A.**

| **Hypothetically admitted (N, %)** | **Work experience** | |  |
| --- | --- | --- | --- |
|  | Residents and ≤10 years (n=71) | >10 years (n=42) | P |
| Case 1 Case 2 Case 3 Case 4 Case 5 | 68 (94)  49 (68)  55 (76)  71 (98)  70 (97) | 40 (97)  31 (76)  30 (73)  40 (98)  41 (100) | 0.89 0.72 0.25 0.13 0.71 |

**B.**

| **Hypothetically admitted (N, %)** | **Practice** | | |  |
| --- | --- | --- | --- | --- |
|  | Academic hospital (n=22) | Top clinical teaching hospital (n=71) | General hospital (n=20) | P |
| Case 1 Case 2 Case 3 Case 4 Case 5 | 22 (100) 16 (73)  17 (77)  22 (100)  21 (95) | 66 (93)  49 (69)  54 (76)  71 (100)  70 (99) | 20 (100)  15 (75)  14 (70)  18 (90)  20 (100) | 0.21 0.28 0.67 0.06 0.49 |

Appendix 4: *The Relationship between the respondent’s work experience and factors influencing decision-making.*

|  | **Case 1** | | **Case 2** | | **Case 3** | | **Case 4** | | **Case 5** | |
| --- | --- | --- | --- | --- | --- | --- | --- | --- | --- | --- |
|  | ***Work experience*** | | ***Work experience*** | | ***Work experience*** | | ***Work experience*** | | ***Work experience*** | |
|  | Residents and ≤10 years (n=71) | >10 years  (n=42) | Residents and ≤10 years (n=71) | >10 years  (n=42) | Residents and ≤10 years (n=71) | >10 years  (n=42) | Residents and ≤10 years (n=71) | >10 years  (n=42) | Residents and ≤10 years (n=71) | >10 years  (n=42) |
| Older age | 21 (20) | 20 (49) | 15 (21) | 10 (24) | 52 (72) | 31 (75) | 38 (53) | 25 (61) | 25 (35) | 18 (43) |
| Antithrombotic use | 69 (97) | 40 (98) | 37 (55) | 28 (59) | 39 (55) | 60 (59) | 50 (70) | 31 (74) | 69 (97) | 40 (95) |
| Support system | 40 (55) | 30 (73) | 31 (43) | 23 (56) | 55 (76) | 32 (78) | 37 (51) | 31 (75) | 28 (38) | 28 (68) |
| Extra-cranial injury | 33 (47) | 28 (67) | 38 (53) | 23 (56) | 34 (47) | 26 (63) | 24 (33) | 23 (56) | 19 (26) | 19 (46) |
| Hemorrhage characteristics | 51 (70) | 28 (68) | 49 (68) | 31 (76) | 52 (73) | 28 (67) | 65 (92) | 37 (88) | 59 (83) | 35 (83) |
